# Membrane transport systems and the biodegradation potential and pathogenicity of genus *Rhodococcus*

**DOI:** 10.3389/fphys.2014.00133

**Published:** 2014-04-04

**Authors:** Carla C. C. R. de Carvalho, Sofia S. Costa, Pedro Fernandes, Isabel Couto, Miguel Viveiros

**Affiliations:** ^1^Department of Bioengineering, Centre for Biological and Chemical Engineering, Institute of Biotechnology and Bioengineering, Instituto Superior Técnico, Universidade de LisboaLisboa, Portugal; ^2^Grupo de Micobactérias, Unidade de Microbiologia Médica, Instituto de Higiene e Medicina Tropical, Universidade Nova de LisboaLisboa, Portugal; ^3^Centro de Recursos Microbiológicos, Universidade Nova de LisboaCaparica, Portugal; ^4^Centro de Malária e Outras Doenças Tropicais, Instituto de Higiene e Medicina Tropical, Universidade Nova de LisboaLisboa, Portugal

**Keywords:** rhodococci, efflux pumps, solvents, antimicrobials, efflux inhibitors

## Abstract

The *Rhodococcus* genus contains species with remarkable ability to tolerate toxic compounds and to degrade a myriad of substrates. These substrates have to cross a distinctive cell envelope dominated by mycolic acids anchored in a scaffold of arabinogalactan covalently attached to the cell wall peptidoglycan, and a cellular membrane with phospholipids, whose composition in fatty acids can be rapidly altered in response to environmental conditions. The hydrophobic nature of the cell envelope facilitates the entrance of hydrophobic molecules but some substrates require active transport systems. Additionally, toxic compounds may also be extruded by energy spending efflux systems. In this review, physiological evidences of the use of transport systems by *Rhodococcus* strains and genomic studies that corroborate their existence are presented and discussed. The recently released complete genomes of several *Rhodococcus* strains will be the basis for an *in silico* correlation analysis between the efflux pumps present in the genome and their role on active transport of substrates. These transport systems will be placed on an integrative perspective of the impact of this important genus on biotechnology and health, ranging from bioremediation to antibiotic and biocide resistance.

## Introduction

### The *Rhodococcus* genus and its taxonomy

The genus *Rhodococcus* comprises aerobic, Gram-positive and non-motile bacterial cells containing mycolic acids. The complex phylogenetic structure of this genus and the difficulty in identifying the different species are emphasized by its long taxonomic history. The name *Rhodococcus* was initially proposed by Zopf (1891) for two red bacteria described by Overbeck as *Micrococcus erythromyxa* and *M. rhodochrous* (Overbeck, 1891; Zopf, 1891). Although the genus *Rhodococcus* was recognized in the editions of 1923–1934 of *Bergey's Manual of Determinative Bacteriology*, strains assigned to the rhodochrous complex were suggested as belonging to several other genera until Tsukamura (1974) revived the genus *Rhodococcus* to which six species previously assigned to the genus *Gordona* were added. In 1977, a more comprehensive numerical taxonomic study provided a better description of the *Rhodococcus* genus and recognized nine species including *Rhodococcus erythropolis* (Goodfellow and Alderson, 1977). Thirty species were listed in the genus *Rhodococcus* in the 2nd edition of *Bergey's Manual of Systematic Bacteriology* (Jones and Goodfellow, 2012).

Based on polyphasic taxonomic data that have been published, members of the *Rhodococcus* genus are placed in the mycolic-acid-forming sub-order *Corynebacterineae*, family *Nocardiaceae*, phylum Actinobacteria*.* The most important characteristics for bacterial cells to be placed in this genus are the following: (i) cell walls containing peptidoglycan consisting of only *meso*-diaminopimelic acid as the diamino acid and arabinose and galactose as major sugars; (ii) mycolic acids containing 30–54 carbon atoms, up to three double bonds and mainly straight-chain saturated, unsaturated and 10-methyl (tuberculostearic)-branched fatty acids; (iii) a phospholipid profile containing diphosphatidylglycerol, phosphatidylethanolamine, phosphatidylinositol, and phosphatidylinositol mannosides, (iv) dehydrogenated menaquinones with eight isoprenoid units, and (v) a high content of G+C in the DNA (Collins et al., 1977; Finnerty, 1992; Bell et al., 1998; Goodfellow et al., 1998; Nishiuchi et al., 2000; Sutcliffe et al., 2010; Jones and Goodfellow, 2012).

Several *Rhodococcus* species are very interesting because of their metabolic plasticity. Their oxidative metabolism is capable of using several organic compounds as sole carbon and energy sources that fostered several industrial and bioremediation applications (Warhurst and Fewson, 1994; Bell et al., 1998; Oldfield et al., 1998; de Carvalho and da Fonseca, 2005a; Larkin et al., 2005). The most successful industrial application of *Rhodococcus* spp. is probably the production of acrylamide by the Nitto Chemical Industry, Co. in Japan (Hughes et al., 1998; Raj et al., 2008; Tao et al., 2009). *Rhodococcus* strains are able to degrade and/or convert highly recalcitrant compounds including aliphatic-, monoaromatic-, and polyaromatic hydrocarbons, as well as heterocyclic aromatic compounds making them suitable in biocatalytic and bioremediation processes (de Carvalho et al., 2007; Pieper and Seeger, 2008; Martínková et al., 2009; Tyagi et al., 2011). They are also potentially pathogenic with some strains causing infections in immunosuppressed patients (Topino et al., 2010; Savini et al., 2012) and in horses (Meijer and Prescott, 2004; Muscatello et al., 2007). Curiously, a gene cluster involved in cholesterol catabolism in *Rhodococcus jostii* RHA1 was found to be conserved in related pathogenic actinomycetes, including *Mycobacterium tuberculosis* (van der Geize et al., 2007; Yam et al., 2011). In fact, genomic analyses have shown that *Rhodococcus* spp. may be useful models for mycobacterial studies: *ca.* 60% of the genes of *M. tuberculosis* strain H37Rv are conserved in *R. jostii* RHA1 (McLeod et al., 2006; van der Geize et al., 2007).

### The biotechnological advantages of the *Rhodococcus* genus

Rhodococci are able to degrade a wide range of hydrophobic natural compounds and xenobiotics such as short-chain, long-chain, and halogenated hydrocarbons, and aromatic compounds, like polycyclic aromatic hydrocarbons, polychlorinated biphenyls and dibenzothiophenes (DBTs) (Larkin et al., 2005; de Carvalho and da Fonseca, 2005b). Their well-established cellular resistance and metabolic ability for the degradation of all these compounds are related to their genomic properties, with an uncommon presence of multiple homologs of enzymes participating in major catabolic pathways and also a remarkable capacity for acquiring large linear plasmids (van der Geize and Dijkhuizen, 2004; Larkin et al., 2005). The ability of rhodococci to degrade substituted hydrocarbons and other chemicals has been used to promote the bioremediation of such compounds in contaminated environments (Sikkema et al., 1995; de Carvalho and da Fonseca, 2005a; de Carvalho et al., 2009; Tyagi et al., 2011). These cells can persist in the soil under nutrient starvation conditions without affecting the breakdown-rate of the pollutants, even if more easily degradable carbon sources are present (Warhurst and Fewson, 1994; Fanget and Foley, 2011). Many of the applications of *Rhodococcus* in industrial interesting processes use resting cells resuspended in medium where the reaction of interest will take place, including the biodesulfurization (BDS) of oil (Caro et al., 2007) and the production of terpenoids (de Carvalho and da Fonseca, 2002, 2003b).

The degradation of hydrophobic pollutants is favored by the very hydrophobic character of *Rhodococcus* cells, which is mainly the result of the presence of aliphatic chains of mycolic acids on the cell wall. The degradation of hydrophobic pollutants in the oil/water interfaces is thus very effective because of a remarkable partitioning of *Rhodococcus* cells into the oil phase (Neu et al., 1992; de Carvalho and da Fonseca, 2003a; Martínková et al., 2009; de Carvalho, 2012). *R. erythropolis* DCL14 cells are even able to change their surface net charge to positive values when growing on long-chain alkanes, which facilitate adhesion to negatively charged surfaces in the environment, such as hexadecane droplets that have a negative zeta potential in aqueous medium (de Carvalho et al., 2009).

Another remarkable aspect of rhodococci is their ability to produce biosurfactants in response to the presence of hydrophobic compounds, such as liquid hydrocarbons. The cellular surfactants produced are predominantly glycolipids (Lang and Philp, 1998) and they promote the bioavailability of compounds with low solubility in water. Biosurfactants also decrease the interfacial tension between organic-aqueous phases, allowing an easier access of hydrophobic compounds to the cells. The dispersion of the hydrophobic compounds caused by the surfactants thus increases the surface area and enhances microbial action (Bell et al., 1998). Therefore, these bacteria are particularly useful for the treatment of chemical wastes by bioremediation (Kosaric, 1992; Desai et al., 1994; Karanth et al., 1999; Banat et al., 2010). Biosurfactants produced by *R. erythropolis*, *R. opacus*, and *R. ruber* can be successfully applied in the oil industry, e.g., for cleaning oil tanks or removing oil from contaminated sands (Ivshina et al., 1998) and also to enhance oil recovery (Pacheco et al., 2010). Strain *R. erythropolis* DCL14 produces a glycolipid biosurfactant in the presence of long-chain alkanes resulting in the reduction of the surface tension of the medium to ca. 23 mN/m (de Carvalho et al., 2009). Adding to this remarkable pleiotropic metabolic behavior, some *Rhodococcus* species are psychrotrophic and/or are able to endure desiccation conditions, which makes this genus important for bioremediation in cold (Yagafarova and Skvortsova, 1996; Belousova and Shkidchenko, 2004) and/or arid climates (Pucci et al., 2000). The adaptation abilities of these cells have also been used to adapt a mesophilic strain to extreme conditions of temperature and pH and to the presence of high amounts of sodium chloride and copper sulfate (de Carvalho, 2012).

Industrial wastes containing toxic compounds such as chlorinated and aromatic hydrocarbons, nitroaromatics and chlorinated polycyclic aromatics can be easily degraded by members of the *Rhodococcus* genus (Bell et al., 1998; de Carvalho and da Fonseca, 2005a; Martínková et al., 2009). Polychlorinated biphenyls (PCBs), which are persistent organic pollutants, are efficiently degraded by *R. rhodochrous* (Boyle et al., 1992) and *R. globerulus* (Asturias and Timmis, 1993). Atrazine and s-triazine containing wastes can be cleaned by *R. corallinus* (Arnold et al., 1996) and crude-oil contaminated environments can be efficiently remediated by e.g., *R. ruber* and *R. erythropolis* (Bell et al., 1998; de Carvalho and da Fonseca, 2005a). The strain *R. erythropolis* DCL14 was described as able to degrade a wide range of toxic compounds, such as *n*-alkanes and aromatic compounds, fuel oil, and motor oil, even under saline and extreme conditions (de Carvalho and da Fonseca, 2005a; de Carvalho et al., 2005, 2007; de Carvalho, 2012). A variety of other recalcitrant, toxic pollutants have also been shown to be degraded by rhodococci such as sulphonated azo dyes; pesticides; carbamates (Bell et al., 1998; de Carvalho and da Fonseca, 2005b); and chlorinated phenols, which are refractory to degradation and the most hazardous and persistent pollutants in soil and groundwater (Haggblom et al., 1989; Briglia et al., 1996; Duque et al., 2012). *Rhodococcus* spp. are also capable of catalyzing the BDS of coal and petroleum, being able to desulfurize refractory organosulfur compounds, which are difficult to desulfurize by conventional chemical-based technologies in the oil industry. BDS using *Rhodococcus*, besides being a promising biotechnological process, requires less energy while preventing sulfurous emissions (Kayser et al., 1993; Davoodi-Dehaghani et al., 2010; Abin-Fuentes et al., 2013).

Several commercially interesting products such as acrylamide, acrylic acid and various amides have been produced by *Rhodococcus* species (Yamada and Kobayashi, 1996; Bell et al., 1998; Bunch, 1998; Abin-Fuentes et al., 2013), as well as dietary supplements and pharmaceuticals, including vitamins such as nicotinamide and para-aminobenzoic acid and the antimycobacterial agents isonicotinic acid hydrazide (isoniazid) and pyrazinamide (Yamada and Kobayashi, 1996). Another interesting biotechnological usage of *Rhodococcus* cells takes advantage of their cholesterol oxidases for the food industry or for the production of steroid drugs (Kreit et al., 1994; van der Geize and Dijkhuizen, 2004; Yam et al., 2011). With high yields and specificity, these bioconversions using different species of *Rhodococcus* have considerable potential for industrial application and have been well explored since 1990.

There are two species with clear interest for biotechnological purposes: *R. rhodochrous* and *R. erythropolis*. The latter is one of the most well studied species of *Rhodococcus* due to its broad substrate specificity, biotechnological properties, and adaptability to extreme conditions (de Carvalho, 2012). These bacteria tolerate, at relative high concentrations, water-miscible solvents such as ethanol, butanol, and dimethylformamide (DMF) (de Carvalho et al., 2004; Yam et al., 2011), and water-immiscible solvents such as long-chain alkanes, aromatic compounds, and phthalates, being highly versatile bacteria for bioremediation and biotransformation processes (de Carvalho and da Fonseca, 2005b; de Carvalho et al., 2007, 2009; Yam et al., 2011).

### The pathological traits of the *Rhodococcus* genus

*Rhodococcus* are ubiquitous: rhodococcal strains have been isolated from samples collected in cold Arctic and Antarctic soil (Whyte et al., 2002), arid sites (Pucci et al., 2000), deep-sea (Colquhoun et al., 1998), animal, and plant tissues (Goodfellow, 1992; de Carvalho and da Fonseca, 2005b). A few of these species can cause infections. *R. fascians* is a phytopathogenic bacterium that causes fasciation in a wide range of monocotyledonous and dicotyledonous plants, an infection that causes several malformations, ranging from deformation of leaves, to witches' brooms formation, and leafy galls (Crespi et al., 1992; Vereecke et al., 2000). Infections caused by *R. fascians* affect a wide range of crops and plants, such as peas and tobacco, causing a significant economic impact (Bell et al., 1998). Although the bacterial infection does not affect the lifespan of the infected plants, the ornamental industry that is based on the plant esthetics is the most affected industry, with considerable financial losses due to infections by *R. fascians* (Depuydt et al., 2008).

*R. equi* (formerly *Corynebacterium equi*) is the etiological agent of the rhodococcosis, a chronic granulomatous pneumonia causing lung abscesses that occurs in horses and other animals, with high incidence in foals under 6 months old (Prescott, 1991; Bell et al., 1998). Although *R. equi* infections may occur in adult horses, foals are the only animals in which infection is common. The symptoms include fever and general respiratory distress. Usually, chronic pus-filled lung abscesses develop and untreated lesions can progress and cause death by asphyxiation (Lavoie et al., 1994). The infection can disseminate from the lungs to the gut lining (causing diarrhea), to other organs and to the joints. Vertebral osteomyelitis can also occur (Prescott, 1991; Bell et al., 1998). Because the bacteria are widespread in soil the herbivore dung provides a good culture medium for the bacterium to grow, keeping foals in crowded conditions may increase the likelihood of exposure to an infective dose of *R. equi* (McNeil and Brown, 1994). Although foals are the most affected mammals by this bacterium, the tuberculosis-like lesions caused by *R. equi* may also occur in cattle and pigs, being the submandibular and other lymph nodes the most frequent lesions present in these mammals (Prescott, 1991; Bell et al., 1998; Gyles et al., 2012).

*R. equi* is a well-known opportunistic agent of infection in patients co-infected with human immunodeficiency virus (HIV) or organ transplanted patients (Harvey and Sunstrum, 1991; Prescott, 1991; Topino et al., 2010; Savini et al., 2012). Similarly to equine infections, human infections are usually located in the lung, causing pneumonia and abscesses, with associated fever, cough, and chest pain (McNeil and Brown, 1994; Topino et al., 2010). However, the infection can disseminate and cause lesions in other organs or bacteraemia becoming often fatal, both in AIDS and non-AIDS immunosuppressed patients (Prescott, 1991; McNeil and Brown, 1994; Topino et al., 2010). Even with an early diagnostic and appropriate treatment, mortality rates in AIDS patients can be high (Topino et al., 2010). Treatment of *R. equi* infections can be difficult, requiring judicious choice of combination of antibiotics and prolonged therapy to avoid relapse (Topino et al., 2010). Furthermore, antibiotics effective *in vitro* against *R. equi* cells may not be effective *in vivo*. *R. equi* is susceptible to several classes of antibiotics, such as macrolides, rifamycins, fluoroquinolones, aminoglycosides, glycopeptides, like vancomycin, and imipenem. The bacterium shows a variable susceptibility to cotrimoxazole, tetracycline, chloramphenicol, clindamycin, and cephalosporins and it is commonly resistant to beta-lactams with some acceptable susceptibility to imipenem (Prescott, 1991; Weinstock and Brown, 2002). Strains of *R. equi* resistant to ciprofloxacin, rifampin, and macrolide antibiotics have already been reported (Giguère et al., 2010; Niwa and Lasker, 2010; Riesenberg et al., 2014).

The tolerance and adaptation of *Rhodococcus*, specially *R. erythropolis*, to several different and aggressive conditions, like the presence of the toxic compounds toluene, carveol, and carvone or heavy-metals and antibiotics (de Carvalho et al., 2005, 2007; Hara et al., 2010; Riesenberg et al., 2014) has been related to the high complexity and capacity of modification, under stress conditions, of the fatty acid composition of the cell membrane, as well as to the usage of putative intrinsic or acquired transport systems (de Carvalho et al., 2005; McLeod et al., 2006; Hara et al., 2010). Since bacteria have the ability to use several mechanisms of defense against hostile environments, it is important to know and enlighten the mechanisms associated to the tolerance, adaptation and resistance of *R. erythropolis*. From the several adaptive mechanisms displayed by bacteria, efflux systems have not, to the best of our knowledge, been described in sufficient detail for rhodococci (de Carvalho et al., 2009; de Carvalho, 2012).

## Transport systems present in the *Rhodococcus* genus

Membrane transport systems are present in both bacterial and eukaryotic cells, participating in key cell functions such as the uptake of essential nutrients, secretion of metabolites, extrusion of noxious byproducts, and maintenance of cellular homeostasis by regulation of the intracellular concentrations of ions and solutes (Saier, 2000). It is predicted that these systems constitute 3–16 % of the total number of open reading frames (ORFs) in prokaryotic genomes (Ren and Paulsen, 2007). Overall, the substrates of such transport systems are water-soluble molecules that will not traverse the cell membrane by simple diffusion, including metals, sugars, amino acids, peptides, oligosaccharides, and macromolecules, such as proteins and nucleic acids (Saier, 2000).

Transporter systems are currently classified according to their function and molecular phylogeny into seven classes, namely Class 1, channels and pores; Class 2, electrochemical potential-driven transporters (including secondary active transporters); Class 3, primary active transporters; Class 4, group translocators; Class 5, transport electron carriers; Class 8, accessory factors involved in transport; Class 9, incompletely characterized transport systems (Saier, 2000; Saier et al., 2009).

The focus of this review is on the transporter systems of the above classes that present the capability to extrude from the rhodococcal cell toxic compounds including solvents, antibiotics and biocides, which are generally designated as efflux pumps. These efflux pumps can be specific, extruding one compound or a class of compounds or multidrug resistance (MDR) efflux pumps that extrude multiple classes of toxic compounds. These MDR transporters can belong to the primary transporters superfamily adenosine triphosphate (ATP)-binding cassette (ABC) that couples the hydrolysis of ATP to the substrate translocation (Class 3) or to the secondary transporter families major facilitator superfamily (MFS), resistance-nodulation-division (RND) superfamily, multidrug and toxic compound extrusion (MATE) family and small multidrug resistance (SMR) that use the proton motive force or the membrane sodium gradient (MATE transporters only) to drive the extrusion of their substrates (Class 2) (Paulsen et al., 1996).

In the current era of genomics, rhodococci have generated some interest due to their wide biotechnological applications and 26 genome sequencing projects are currently ongoing, of which five are already completed and represent four species: *R. erythropolis*, *R. equi*, *R. opacus*, and *R. jostii* (National Center for Biotechnology Information, 2005). The analysis of these genomes revealed different structures, *R. equi* and *R. erythropolis* present smaller circular genomes (5 and 6.9 Mb) whereas *R. opacus* and *R. jostii* have larger linear chromosomes (8.8 and 9.7 Mb) (National Center for Biotechnology Information, 2005; McLeod et al., 2006; Letek et al., 2010; Pathak et al., 2013; Shevtsov et al., 2013). Another characteristic of rhodococcal genomes is the common presence of plasmids, one to five per strain, either circular or linear, that range in size from 3 kb to more than 1 Mb, accounting for up to 20% of the entire genome (e.g., *R. jostii* strain RHA1) (Letek et al., 2008, 2010). Despite these differences, the ratio of transporter systems per Mb of genome is similar in the sequenced genomes (0.08–0.1), although some differences are encountered when analyzing the overall number of transporters (Table 1).

**Table 1:** **Distribution of the families of transporter systems in the genomes of four distinct *Rhodococcus* species (Transporter classification database, Saier et al., 2006; TransportDB, Ren et al., 2007)**.

**Organism**	**Genome size (Mb)**	**Number of transporters /Mb genome**	**Transporter type (%)**				
			**ATP-dependent**	**Ion channels**	**PTS**	**Secondary transporters**	**Unclassified**
*R. equi* 103S	5.043	0.09	54.9	3.4	n.i.	38.5	3.2
*R. erythropolis* PR4	6.896	0.10	55.7	2.2	1.0	39.4	1.7
*R. opacus* B4	8.835	0.09	47.1	2.1	0.8	48.1	1.9
*R. jostii* RHA1	9.703	0.08	46.5	2.1	0.7	48.6	2.1

ATP, adenosine triphosphate; PTS, phosphotransferase systems n.i., not identified.

An *in silico* comparative analysis predicted the occurrence of MDR efflux pumps in the genomes of these rhodococci, ascribed to several transporter families. Of relevance is the prediction of the presence of structurally complex and multipartite RND superfamily MDR efflux pumps in this gram positive genus. However, a main constraint of this analysis is the lack of *in vivo* data supporting the transporters classification, either in rhodococci or in other related genera. A second constraint is the low identity observed for the putative efflux pumps identified among the several rhodococcal species, with identity values of 65–75% for the majority of the transporters, with an exception for *R. jostii* RHA1 and *R. opacus* B4 that present high identities (up to 99%). These low identity values are also registered when comparing putative efflux pumps from rhodococci and related genera, such as *Corynebacterium* spp. and *Mycobacterium* spp.

BLAST analysis of the putative MDR efflux pumps identified, which are mainly chromosomally-encoded, revealed a similar number of transporters of the different families in the four genomes analyzed, namely the occurrence of 11–15 ABC efflux pumps, 50–70 MFS transporters, 5–9 pumps of the RND superfamily, 1–3 SMR efflux pumps and 1 MATE transporter. In Table 2 are presented the MDR efflux pumps for which a high identity (>75%) was found among the four rhodococcal species in analysis.

**Table 2:** ***In silico* prediction of chromosomal MDR transporter systems involved in resistance to antimicrobial agents in representative rhodococcal species (sharing > 75% identity) and their relation to *Mycobacterium tuberculosis* H37Rv transporters**.

Type of transporter	Rhodococcal strain				
	***R. equi* 103S**	***R. erythropolis* PR4**	***R. jostii* RHA1**	***R. opacus* B4**	***M. tuberculosis* H37Rv**
**PUTATIVE ABC TRANSPORTER INTEGRAL MEMBRANE SUBUNIT**					
	REQ_11220	RER_56560 (71%)	RHA1_ro02603 (69%)	ROP_03190 (77%)	–
	REQ_12170	RER_43530 (84%)	–	–	–
	REQ_20440		RHA1_ro06840 (83%)	ROP_68250 (83%)	–
	REQ_24790	RER_32730 (77%)	RHA1_ro00945 (80%)	ROP_06760 (80%)	Rv1686c (64%)
	REQ_30790	RER_46100 (75%)	RHA1_ro02603 (50%)		–
		RER_44380	RHA1_ro04792 (88%)	ROP_48830 (88%)	–
**PUTATIVE ABC TRANSPORTER INTEGRAL MEMBRANE/ATPase SUBUNITS**					
	REQ_03250	RER_03410 (72%)	RHA1_**r**o04169 (79%)	ROP_40980 (79%)	–
	REQ_14720	RER_41210 (74%)	RHA1_ro06013 (78%)	ROP_60720 (78%)	Rv0194 (43%)
			RHA1_ro01004	ROP_07300 (97%)	Rv1819c (52%)
**PUTATIVE MFS TRANSPORTER**					
	REQ_00410		RHA1_ro05096 (75%)	ROP_51570 (77%)	–
	REQ_08920	RER_55050 (75%)			Rv1634 (53%)
	REQ_09900	RER_46140 (79%)	RHA1_ro04995 (78%)	ROP_50560 (80%)	–
	REQ_13270	RER_55220 (78%)			–
	REQ_15920		RHA1_ro01594 (76%)	ROP_12980 (77%)	Rv1258c (55%)
	REQ_18780		RHA1_ro06618 (75%)	ROP_66540 (75%)	EfpA (53%)
	REQ_19100		RHA1_ro06636 (83%)	ROP_66710 (83%)	–
	REQ_20430		RHA1_ro05369 (80%)	ROP_54620 (80%)	–
	REQ_23090	RER_31910 (75%)	RHA1_ro00861 (75%)	ROP_05990 (75%)	–
	REQ_27500	RER_35250 (77%)	RHA1_ro01063 (78%)	ROP_07910 (77%)	–
	REQ_30590	RER_38560 (80%)	RHA1_ro01413 (83%)	ROP_11240 (84%)	Rv_2508c (51%)
	REQ_36620	RER_17440 (75%)	RHA1_ro01949 (76%)		Rv_0783c (51%)
	REQ_38480	RER_15430 (79%)			–
	REQ_39850	RER_13960 (85%)	RHA1_ro05519 (87%)	ROP_55930 (87%)	–
	REQ_45350		RHA1_ro04268 (79%)	ROP_41810 (79%)	–
		RER_13470	RHA1_ro04142 (79%)	ROP_40760 (79%)	–
		RER_19430	RHA1_ro05093 (83%)	ROP_51550 (82%)	Rv2459 (35%)
		RER_39310	RHA1_ro02360 (82%)	ROP_20740 (85%)	–
		RER_55700	RHA1_ro04268 (78%)	ROP_41810 (78%)	–
**PUTATIVE SMR PROTEIN**					
	REQ_07770	RER_09840 (81%)	RHA1_ro04738 (80%)	ROP_48320 (80%)	–
**PUTATIVE MATE EFFLUX FAMILY PROTEIN**					
	REQ_19210	RER_26360 (74%)	RHA1_ro06648 (78%)	ROP_66870 (77%)	Rv_2836c (63%)
**PUTATIVE RND FAMILY PROTEIN**					
mmpL1	REQ_03240	RER_03400 (78%)	RHA1_ro04168 (80%)	ROP_40970 (79%)	–
mmpL2	REQ_22980	RER_31970 (77%)	RHA1_ro03267 (79%)	ROP_27830 (80%)	–
mmpL3	REQ_34640	RER_09760 (74%)	RHA1_ro11238 (74%)	ROP_00400 (76%)	MmpL11 (30%)
mmpL4	REQ_34730		RHA1_ro06222 (73%)	ROP_62810 (73%)	Rv0206c (40%)
mmpL6	REQ_44510	RER_52340 (76%)	RHA1_ro02326 (78%)	ROP_20440 (77%)	–

The identity percentage shown was calculated relatively to R. equi 103S or R. erythropolis PR4 genomes.

The study of drug resistance efflux systems in the related genus *Mycobacterium* is still in early stages, compared to other bacteria. Nevertheless, some efflux pumps were described and experimental data gathered supporting their role in drug resistance (De Rossi et al., 2006; Louw et al., 2009; Viveiros et al., 2012; Machado et al., 2012). Genome analysis showed the presence of rhodococcal transporters with significant identity to those efflux pumps (Table 2). For example, the MFS efflux pump Rv1258c from *M. tuberculosis* H37Rv, associated with the export of the antibiotics isoniazid, rifampicin, ethambutol, and ofloxacin (Louw et al., 2009; Machado et al., 2012), has ca. 55% identity to the transporters REQ_15920, RHA1_ro01594, and ROP_12890 of *R. equi* 103S, *R. jostii* RHA1, and *R. opacus* B4, respectively.

Putative MFS MDR transporters are also found in the rhodococcal plasmids sequenced so far; two in the linear plasmid pREL1 (272 kb) of *R. erythropolis* PR4, and several in the linear plasmids of *R. jostii* RHA1, namely two in pRHL1 (330 kb), six in pRHL2 (440 kb), and one in pRHL3 (1.1 Mb).

Specific efflux pumps also occur in the rhodococci genomes, with a substrate specificity mainly devoted to the transport of heavy-metal ions, such as arsenite and cadmium, a clear reflection of the environmental niches that these species inhabit (Table 3). Another particular characteristic is their presence in rhodococcal plasmids. A large number of these plasmids are conjugative or mobilizable (Letek et al., 2010) carrying several niche-specific genes, including the pathogenicity island PAI, which harbors the *vap* genes, such as *vapA* that encodes a surface antigen in *R. equi* (Takai et al., 2000). Plasmid-mediated resistance to heavy-metals was first reported in a strain of *R. erythropolis*. The plasmids identified carried determinants conveying resistance to arsenate, arsenite, and cadmium (Dabbs and Sole, 1988). In the same year, plasmid-borne cadmium resistance was also described for the phytopathogenic *R. fascians* (Desomer et al., 1988). In both studies, co-localization of a determinant encoding resistance to the antibiotic chloramphenicol was also observed in some plasmids (Dabbs and Sole, 1988; Desomer et al., 1988). Following studies on the 160 kb conjugative plasmid pRF2 of *R. fascians* revealed the chloramphenicol-resistance determinant *cmr* as coding for a MFS transporter with 12 transmembrane segments (Desomer et al., 1992). A *cmr*-like gene, *cmrA*, encoding the CmrA protein with 86% amino acid identity to Cmr, was also found on a plasmid of *R. rhodochrous* (Quan and Dabbs, 1993; De Mot et al., 1997). A putative tetracycline specific MFS efflux pump, RHA1_ro08008, is also found in the *R. jostii* RHA1 linear plasmid pRHL1 (1.1 Mb). BLAST analysis of this putative Tet pump revealed homology (86% identity) to a MFS transporter in *R. opacus* B4 chromosome (ROP_04480).

**Table 3:** **List of putative specific transporters for heavy-metals present in representative rhodococcal species**.

**Substrate**	**Transporter**	**Family**	**Species (No. of transporters)**	**Gene location (No. of transporters)**
Arsenite	ArsA	Arsenite-antimonite (ArsAB) efflux family	*R. equi* (1)	C
			*R. erythropolis* (4)	C (3) + P (1)
			*R. jostii* (3)	C
			*R. opacus* (4)	C
	ArsB		*R. equi* (1)	C
			*R. erythropolis* (1)	C
			*R. jostii* (1)	C
			*R. opacus* (1)	C
	UN	Arsenical resistance-3 (ACR3) family	*R. equi* (1)	C
			*R. erythropolis* (2)	C (1)+ P (1)
			*R. jostii* (1)	C
			*R. opacus* (3)	C
Cadmium	UN	P-type ATPase	*R. jostii* (1)	C
Cobalt	UN	ATP-Binding cassette (ABC)	*R. equi* (8)	C
			*R. erythropolis* (6)	C
			*R. jostii* (5)	C
			*R. opacus* (5)	C
Cobalt/Zinc/Cadmium	UN	P-type ATPase	*R. equi* (4)	C
			*R. erythropolis* (5)	C (3) + P (2)
			*R. jostii* (5)	C
			*R. opacus* (3)	C

UN, unnamed; C, chromosome; P, plasmid.

Determinants for resistance to heavy-metals are widely disseminated in rhodococcal plasmids (Table 3). For instance, the linear plasmid pREL1 from *R. erythropolis* PR4 harbors several heavy-metal transporters, including predicted cadmium-efflux P-type ATPases, a cadmium transporter from the *cadD* family and an arsenite transporter from the ACR3 family (Sekine et al., 2006). The characterization of the catabolic plasmids pRHL3 from *R. jostii* RHA1 and pDB2 from *R. erythropolis* BD2 (210 kb) showed the presence of several transporters involved in the export of the metal ions arsenate, arsenite, cadmium, copper and mercury (Dabrock et al., 1994; Stecker et al., 2003; Warren et al., 2004). Heavy-metal transporters can also occur in the chromosome and many are substrate promiscuous transporting many different classes of compounds.

## Transport of solvents, antibiotics, and biocides

As mentioned before, rhodococci are well known for thriving under harsh environments, where few other microorganisms are able to endure. Such behavior has been mostly related to changes in the cell wall, that hinders the diffusion of deleterious compounds; or to the metabolic versatility displayed by members of this genus, which result in the partial or total mineralization of the toxic compounds or proper processing for use as carbon and/or energy source (Larkin et al., 2005; de Carvalho et al., 2005, 2009; de Carvalho, 2012). Although active transport is a well-known tool for microbial strains to deal with toxic compounds (Torres et al., 2011; Kriszt et al., 2012; Nikaido and Pagès, 2012; Segura et al., 2012) there are very few works where this method has been clearly identified in *Rhodococcus* strains despite this possibility is often suggested. Such exceptions include: (a) the selective transport of n-hexadecane by *R. erythropolis* S+14He (Kim et al., 2002). This particular strain was shown to discriminate n-hexadecane from mixtures of alike molecules and transport it into the cell by an energy driven mechanism, where it could be accumulated and used as carbon source; (b) the uptake of cesium ion through an energy dependent mechanism, which configures its application in the treatment of cesium contaminated environments (Ivshina et al., 2002); (c) the uptake of phthalates by *R. jostii* RHA1, where, unlike in the two former examples, the active influx behavior was specifically ascribed to an ABC transporter, encoded by *patDABC* genes. From bioinformatic analysis with the NCBI Conserved Domains database, the authors were able to suggest the individual roles of the components within the transporter. Thus, PatA was the cytoplasmic ATP-binding component of the transporter, PatB and PatC were membrane-spanning permease components of the transporter and PatD was the extracytoplasmic substrate-binding component of the transporter. The work performed moreover contributed to establish the feasibility of the use of *R. jostii* RHA1 cells as scavengers of the environmentally pollutant phthalates (Hara et al., 2010). The active transport of 4, 6-dimethyldibenzothiophene (DBT) in *R. erythropolis* LSSE8-1 was highly promoted after effective transformation of the wild type strain with the plasmid HcuABC containing the DBT uptake genes of *Pseudomonas delafieldii* R-8 (Wang et al., 2011). This enhanced the transfer of DBT into the cells, resulting in higher desulfurization rates when compared to the wild-type R. *erythropolis* LSSE8-1.

Similarly to *M. tuberculosis*, the mycolic acids of *Rhodococcus* sp. should contribute to a high hydrophobicity of the cell envelope thus hampering the entry of antibiotics to the cell. A relation between antibiotic resistance and lipid composition of rhodococci grown on *n*-alkanes was found in strains of *R. ruber* (Kuyukina et al., 2000). After growth in rich organic media containing gaseous or liquid *n*-alkanes, the cells exhibited increased resistance to aminoglycosides, lincosamides, macrolides, and β-lactams. The challenged *R. ruber* cells increased their lipid content and the percentage of saturated straight-chain fatty acids, while starting to produce cardiolipin and phosphatidylglycerol. The resulting decrease in the permeability of the cell envelope should have been responsible for a decreased penetration of the antibiotics in the cells.

Actinomycetales include species which produce most of antibiotics in use as secondary metabolites (Fiedler et al., 2005; McLeod et al., 2006; Waksman et al., 2010). Biosynthesis of antibiotics has been reported for *Rhodococcus* spp. (Kitagawa and Tamura, 2008). It is thus expected that these cells produce transporters involved in drug export. During a partial genome sequencing of *R. equi* ATCC 33701, no beta-lactamase enzymes or aminoglycoside acetyl transferase genes were identified, but potential drug-efflux systems included 12 efflux protein genes and at least 25 ABC transporter proteins (Rahman et al., 2003). However, the more recent genome analysis of *R. equi* 103S revealed the presence of a wide array of antibiotic resistance determinants, including five aminoglycoside phosphotransferases and 10 beta-lactamases (Letek et al., 2010). *Rhodococcus* sp. RHA1 contains genes encoding a high number of peptide synthetases and polyketide synthases (providing evidence for an extensive secondary metabolism) and a corresponding high number of transporters potentially involved in drug export (McLeod et al., 2006). Resistance to glycopeptides has been reported in *R. equi* human clinical strains (Hsueh et al., 1998) and a novel operon for glycopeptide resistance, *vanO*, with low homology with enterococcal operons, was recently identified in *R. equi* isolated from soil (Gudeta et al., 2014).

As mentioned previously, two efflux exporters of chloramphenicol have been identified in the strains *R. fascians* and *R. rhodochrous*. Also, two putative tetracycline transporters are also present in the *R. jostii* and *R. opacus* genomes. In general, the majority of the MDR efflux pumps present in other bacteria, such as *Escherichia coli* and *Staphylococcus aureus* have the capacity to extrude antibiotics as well as other antimicrobial agents known as biocides (Poole, 2005). Thus, it is possible that the putative MDR efflux pumps present in the several rhodococcal species also share the same capacity of biocide extrusion.

## Inhibition of transport in the *Rhodococcus* genus

Targeting the membrane transport systems is an attractive option to reduce the activity of efflux pumps when retention of a given substrate is desired either because (i) it guarantees more substrate for a biotransformation occurring inside the cell or (ii) it promotes accumulation of a toxic compound inside the cell, allowing it to reach the intended target, as in the case of antibiotics that are subject to efflux by *Rhodococcus* species. The discovery and development of inhibitory molecules and strategies to circumvent efflux are needed. Inhibitors of efflux pumps have been identified through standard high-throughput screening of synthetic chemical libraries of compounds or have been isolated from natural sources known for their ethnomedicinal properties (Pagés et al., 2011; Rodrigues et al., 2011). More recently, molecular modeling combined with crystallography and docking-studies allowed the directed drug design of the best pharmacophoric groups of putative inhibitory molecules that might be involved in substrate recognition and binding within a specific efflux pump, as was the case of spectinamides in *M. tuberculosis*. A new group of antitubercular compounds was created modifying a molecule, spectamycin, to prevent efflux by the Rv1258c pump of *M. tuberculosis* (Lee et al., 2014), the one that has a high identity to transporters present in the genome of different rhodococci. Consequently, the development of antimicrobial agents that are less susceptible to be effluxed or which may interfere with the kinetics of transport, will be a great benefit to revive or reuse many of the drugs that have become ineffective as antimicrobials and can also be of great advantage for biotransformation purposes for genus of the order Actinomycelales (Fernandes et al., 2003; Rodrigues et al., 2011; Torres et al., 2011).

**Figure 1:**
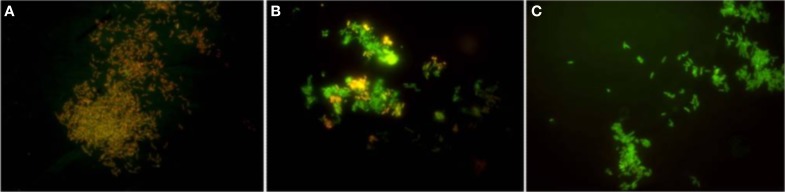
**The temporal [(A) 5 min, (B) 30min, (C) 60 min] effect of the efflux inhibitor verapamil used at a non-lethal concentration on the membrane potential of *R. erythropolis* cells stained by a commercial dye.** Orange/red cells—fully polarized. Green cells—fully depolarized. Adapted from Vencá (2012).

The development of new tools and methodologies that afford the intelligent design of compounds that target distinct properties of a given efflux pump and allow the study of the kinetics of transport is deeply needed. One of the recent developments in this area has been the development of methodologies based on the *in vivo* real-time fluorometric detection of the accumulation and efflux kinetics of fluorescent dyes such as ethidium bromide (Viveiros et al., 2008, 2010), red-Nile (Bohnert et al., 2010), or bisbenzimide (Hoechst 33342) (Coldham et al., 2010). Such methodologies were very helpful for the study of the efflux activity in different mycobacterial species (Viveiros et al., 2008, 2010; Machado et al., 2012). The genomic and physiological similarities between *Mycobacterium* and *Rhodococcus* genus makes this research approach suitable for studies aiming to identify promising targets for effective compounds to control efflux in the *Rhodococcus* genus. Recently, and following this rational, the detection of efflux activity in *R. erythropolis* DCL14 cells, in particular the real-time fluorometric detection of accumulation and efflux of ethidium bromide, a common substrate of MDR efflux pumps, has provided preliminary evidences of efflux activity in this species (Vencá, 2012). Moreover, it has also been shown that this efflux activity can be inhibited by compounds known as inhibitors of efflux pumps in other bacteria, namely *Mycobacterium* spp. (Viveiros et al., 2012), *S. aureus* (Costa et al., 2011) and *E. coli* (Viveiros et al., 2007; Paixão et al., 2009), such as the protonophore carbonyl cyanide m-chlorophenylhydrazone (CCCP) or the calcium channel blockers verapamil and the phenothiazines thioridazine and chlorpromazine (Vencá, 2012). These compounds are known to interfere with the membrane potential of eukaryotic and prokaryotic cells by disrupting or reducing the membrane potential therefore depleting the energy necessary to maintain active transport (Amaral et al., 2007; Viveiros et al., 2008; Rodrigues et al., 2011). An illustrative example of the disruption of *R. erythropolis* membrane potential during 60 min of exposure to a non-lethal concentration of verapamil can be seen in Figure 1.

To be classified as an efflux inhibitor, a compound has to satisfy some basic criteria (Lomovskaya and Watkins, 2001): (i) it must enhance the activity of multiple substrates of the pump by retaining them inside the cells; (ii) it should have no activity in strains that do not have the efflux pump; (iii) it should increase accumulation and decrease efflux of the efflux pump substrates; (iv) its activity must not affect directly the integrity of the bacterial membrane nor the cell viability, at the concentrations used for efflux inhibition. CCCP is the canonical example of an efflux-inhibitor that uncouples the proton gradient established during the normal activity of electron carriers in the electron transport chain, seriously affecting the energy level of the membrane, causing a dissipation of the proton motive force, with a similar effect as the one seen in Figure 1, therefore affecting all the transporters that depend on this energy source to operate (Kašèáková et al., 2012). The same effect can be indirectly achieved by the use of ATPase inhibitors such as sodium orthovanadate, described as an inhibitor of efflux systems dependent on ATPases, like the ABC efflux pumps (Komeda et al., 1997; Garvey and Piddock, 2008; Vencá, 2012).

These powerful, direct or indirect, uncouplers of the proton motive force promote the collapse of the membrane energy and consequently, when used at low concentrations that do not affect the viability of the cell, they inhibit the active transport through the bacterial membrane of efflux substrates such as excretion metabolites, toxic compounds, solvents, biocides, and antibiotics. The inhibitory activity results in the accumulation of the substrate inside the bacterial cell promoting their intracellular activity, either inhibiting the bacterial replication (as in the case of antibiotics and antibiotic resistance) or promoting intracellular biotransformations (as in the case of organic compounds transformation/degradation and solvent tolerance). Controlling this transport phenomenon has important implications on therapeutics and biotransformation as it has been demonstrated by different groups on the closely related *Mycobacterium* genus that we have previously demonstrated the Rhodococci share great affinities (Fernandes et al., 2003; Torres et al., 2011; Viveiros et al., 2012).

From the results obtained so far by different groups, either on the physiological level or the genotypic level, it is evident that rhodococci have the ability to easily gain tolerance to solvents and antimicrobial drugs and the inhibition of their efflux systems may be an effective strategy to improve both the biotechnological and bioremediation properties of these bacteria and also to improve the antimicrobial effectiveness of the few antibiotics that are active against these bacteria. By blocking efflux pumps activity, deleting the efflux pump or controlling the regulatory factors (e.g., sigma factors) that control the efflux pump expression the concentration of the transported substrate retained inside the cells will be higher and its effect may increase (Fernandes et al., 2003; Kita et al., 2009; Segura et al., 2012; Vencá, 2012). This approach leads to the possibility to reduce not only the intrinsic resistance/tolerance but also to reverse the acquired resistance/tolerance of bacteria to drugs and solvents (Fernandes et al., 2003; Viveiros et al., 2008, 2012; Rodrigues et al., 2011). Nothing has been found in the literature about the usage of rhodococci efflux pump knock-out mutants for biotechnological purposes. Noteworthy to mention, reversing tolerance to solvents may not be the desirable biotechnological outcome when bacteria, such as rhodococci, are used for bioremediation purposes: inhibiting efflux pumps in the presence of a toxic pollutant may compromise bacterial viability. What is intended is to optimize the biodegradative characteristics of the bacteria without affecting its viability and to promote the most beneficial aspects of their bioremediation abilities which could contribute to the bioremediation of contaminated sites (Fernandes et al., 2003; de Carvalho et al., 2007; de Carvalho, 2012).

The presence of efflux activity and active efflux pumps in rhodococcal cells together with the several catabolic pathways displayed by *Rhodococcus* spp. clearly suggest an important role of efflux systems in the adaptation of these bacteria to their ecological niches. For example, drug resistance efflux pumps may be crucial to the survival of *Rhodococcus* that are present in the soil, sharing their habitat with several antibiotic producing bacteria, such as *Streptomyces* spp. It should, nevertheless, be pointed out that *Rhodococcus* cells are able to produce large numbers of secondary metabolites and of degrading/converting a wide variety of substrates of substrates. For such compounds to be degraded and/or converted they must be allowed to enter the cell and reach the necessary enzymes. The complex system regulating the entrance/exit of compounds that are able to kill many other bacteria is still far from being elucidated and interfering with efflux is obviously one strategy to control the highly adaptative traits of the *Rhodococcus* genus (de Carvalho and da Fonseca, 2005b; de Carvalho, 2012).

## Conclusion and perspectives

From the evidences gathered in this review it is possible to conclude that the *Rhodococcus* genus is composed by highly adaptable bacteria capable of tolerating high amounts of a wide range of toxic compounds and it has, therefore, interesting applications in biotechnology and bioremediation. The tolerance of rhodococci is clearly associated with its plastic genome coding for numerous efflux pumps, combined with a very versatile metabolism that makes this genus unique in terms of the ability to survive under extreme environmental conditions. This review brings additional insights on the distribution among rhodococcal species of genes involved in active transport and mobilization previously demonstrated and reported in the literature. It is clear that rhodococci specialized in transporting substrates such as solvents and drugs, with some rhodococcal species, such as *R. opacus*, *R. erythropolis*, and *R. jostii*, being well equipped with genes for the transport of many different substrates. This reviews represents a starting point for the development of further studies connecting the catalog of rhodococcal genes involved in active transport, that are currently being unraveled by whole-genome sequencing projects, with their respective panel of substrates and inhibitors, transport kinetics and possibility to control the kinetics for biotechnological purposes.

In the genomic era, the most common methodology to study transporter proteins is still to sequence the bacteria genome and, by comparison of homologous bacterial genomes and the strategy of knocking-out or overexpressing the genes, to find sequences that would probably encode efflux pumps. This approach is further supplemented with the determination and comparison of the inhibitory effects of different substrates against the wild-type and knock-out/overexpressing variants and deducting from the reduction of the susceptibilities to these substrates, a direct connection between the efflux pump and the substrate effluxed. This methodology is not adequate to study substrate transport from a bioengineering perspective and does not allow the determination of transport parameters. Therefore, it is necessary to continue the development of adequate methods to characterize and understand transport phenomena in bacteria, e.g., by detection and quantification of drug and solvent transport across the bacterial cell wall. This would greatly improve the biotechnological and bioremediation applications of this genus. The determination of kinetics and transport parameters should be further explored and these models and parameters will be of great importance to control the bacterial transport machinery in order to optimize their beneficial aspects. From the biomedical point of view, the ability to retain and concentrate antimicrobials inside *Rhodococcus* cells by the use of inhibitors or chemical modifications of substrates to circumvent efflux and reduce tolerance/resistance, will undoubtedly benefit the therapeutics of rhodococcal infections as it is now recognized for mycobacterial infections (Adams et al., 2014; Viveiros and Pieroni, 2014).

The association of efflux activity and adapted/tolerant/resistant phenotypes in the *Rhodococcus* genus, either for biotechnological or biomedical purposes, is nowadays a challenge for science. The functional identification and characterization of the key genes involved in both solvent and antibiotic transport, including those participating in regulatory mechanisms, the creation of knock-out gene mutants for these transport mechanism and the design and/or the identification of new drugs that can overcome efflux in the genus, will be one of the major challenges in this field for the coming future. The sum-up of results gathered in the literature so far clearly indicate that this group of bacteria possesses enough genetic information to deploy a series of physiological strategies to respond to the presence of noxious compounds in the surrounding environment and is able to use a series of transport mechanisms to survive to these external menaces. It is now up to us to learn how to master such mechanisms in order to promote the biodegradation potential and reduce the pathogenicity of the *Rhodococcus* genus.

### Conflict of interest statement

The authors declare that the research was conducted in the absence of any commercial or financial relationships that could be construed as a potential conflict of interest.
